# How Soluble Fms-Like Tyrosine Kinase 1 Could Contribute to Blood-Brain Barrier Dysfunction in Preeclampsia?

**DOI:** 10.3389/fphys.2021.805082

**Published:** 2022-02-08

**Authors:** Pablo Torres-Vergara, Robin Rivera, Carlos Escudero

**Affiliations:** ^1^Department of Pharmacy, Faculty of Pharmacy, Universidad de Concepción, Concepción, Chile; ^2^Group of Research and Innovation in Vascular Health (GRIVAS Health), Chillán, Chile; ^3^Vascular Physiology Laboratory, Department of Basic Sciences, Faculty of Sciences, Universidad del Bío-Bío, Chillán, Chile

**Keywords:** cerebrovascular complications, blood-brain barrier, stroke, VEGF, sFlt-1, preeclampsia

## Abstract

Preeclampsia is a pregnancy-related syndrome that courses with severe cerebrovascular complications if not properly managed. Findings from pre-clinical and clinical studies have proposed that the imbalance between pro- and anti-angiogenic factors exhibited in preeclampsia is a major component of its pathophysiology. In this regard, measurement of circulating levels of soluble tyrosine kinase-1 similar to fms (sFlt-1), a decoy receptor for vascular endothelial growth factor (VEGF), is a moderately reliable biomarker for the diagnosis of preeclampsia. However, few studies have established a mechanistic approach to determine how the high levels of sFlt-1 are responsible for the endothelial dysfunction, and even less is known about its effects at the blood-brain barrier (BBB). Since the expression pattern of VEGF receptors type 1 and 2 in brain endothelial cells differs from the observed in peripheral endothelial cells, and components of the neurovascular unit of the BBB provide paracrine secretion of VEGF, this compartmentalization of VEGF signaling could help to see in a different viewpoint the role of sFlt-1 in the development of endothelial dysfunction. In this article, we provide a hypothesis of how sFlt-1 could eventually be a protective factor for brain endothelial cells of the BBB under preeclampsia.

## Introduction

According to the International Society for Study of Hypertension in Preeclampsia (ISSHP), preeclampsia is defined as the presence of *de novo* hypertension (≥140/90 mmHg) after 20 weeks of gestation accompanied by proteinuria and/or a series of systemic symptoms and/or fetal growth restriction (Brown et al., 2018). Preeclampsia affect 1–5% of pregnancies, and it would be responsible for 70,000–80,000 maternal deaths and 500,000 perinatal deaths worldwide every year (Hutcheon et al., 2011). In Latin America and the Caribbean, hypertensive disorders account for almost 26% of maternal deaths (Khan et al., 2006). Causes of maternal death due to preeclampsia in low- and middle-income countries are mainly associated with cerebrovascular complications (Miller, 2019).

In human, preeclampsia is a pregnancy-related syndrome that requires the presence of a placenta to develop (Redman and Sargent, 2005; Kanter et al., 2010). The two-stage model proposes a poor placentation leads to the placental release of harmful factors into the maternal circulation, resulting in the development of endothelial dysfunction (Roberts et al., 1989; Redman and Sargent, 2005). Under this sequence of events, the cerebrovascular complications of preeclampsia represent an additional example of multisystemic endothelial damage. This model has evolved over the years including factors such as inflammation, angiogenic imbalance and maternal risk factors (Staff, 2019). However, this point of view still seems to be an oversimplification of the pathogenesis of cerebral complications, as approximately one third of the women with eclampsia develop mild hypertension (Sibai, 2012) and cerebral complications are also detected post-partum (Razmara et al., 2014).

Among the circulating placental factors present in the maternal circulation, the soluble truncated vascular endothelial growth factor (VEGF) receptor type 1 or tyrosine kinase-1 similar to fms (sFlt-1), has received increasing attention due to its clinical applicability as risk factor for preeclampsia (Maynard et al., 2003; Levine et al., 2004). High levels of sFlt-1 are thought to lead to an angiogenic imbalance, but despite hundreds of publications focusing on sFlt-1 and preeclampsia, the underlying cellular mechanism by which sFlt-1 generate endothelial dysfunction in this syndrome are poorly known, and even less is known about its potential effects at the blood-brain barrier (BBB).

Physiologically, VEGF binds and activates the tyrosine-kinase receptors VEGF receptor type 1 (VEGFR1), type 2 (VEGFR2), and type 3 (VEGFR3) (Simons et al., 2016). Since sFlt-1 works as a decoy VEGFR with greater binding affinity to VEGF compared to VEGFR2, this function balances the effects of VEGF signaling on vascular development, permeability, and integrity. Therefore, sFlt-1 is an antagonist to VEGFR2 signaling.

Vascular endothelial growth factor receptor type 2 is predominately expressed in vascular endothelial cells, including brain endothelial cells, where activation of VEGFR2 consistently leads to increased permeability of the BBB (Schreurs et al., 2012; Hudson et al., 2014), as observed in pathological conditions including stroke (Li et al., 2014) and multiple sclerosis (Argaw et al., 2012; Chapouly et al., 2015). Several reports have demonstrated that brain endothelial cells become more reactive to the paracrine secretion of VEGF from neurons and astrocytes under pro-inflammatory and hypoxic conditions (Argaw et al., 2012; Li et al., 2014; Chapouly et al., 2015), being this outcome consistent with the higher basolateral expression of VEGFR2 in this cell type, when compared to the observed in peripheral endothelial cells (Hudson et al., 2014). This compartmentalization of VEGF signaling in brain endothelial cells invites to reassess the role of sFlt-1 in the development of brain endothelial dysfunction in preeclampsia. In this article, we provide a hypothesis of how sFlt-1 could eventually be a protective instead of harmful factor for brain endothelial cells of the BBB, and how it may contribute to the pathophysiology of cerebrovascular complications in preeclampsia based on the current state of the art.

## Cerebrovascular Complications in Preeclampsia

Preeclampsia-associated cerebrovascular complications include eclampsia (new onset of seizures in women with preeclampsia), haemorrhagic and ischemic stroke, edema formation, brain herniation, posterior reversible encephalopathy syndrome (PRES), and reversible cerebral vasoconstriction syndrome (RCVS) (Hammer and Cipolla, 2015). These alterations may cause short- and long-term morbidities including white matter injuries (Hecht et al., 2017; Siepmann et al., 2017), higher post-partum cerebrovascular disease risk and maternal death. Cerebrovascular alterations are the direct cause of approximately 40% of maternal deaths (MacKay et al., 2001).

Epidemiologically, the estimated prevalence of stroke during pregnancy and post-partum is 34 per 100,000 deliveries (James et al., 2005), and a study reported that the risk of post-partum stroke within 60 days after delivery in women with pregnancy-related hypertension is 41.7% (Too et al., 2018). However, the exact prevalence of PRES in preeclamptic and eclamptic women is not well known, but a retrospective study found that PRES was present in more than 90% of eclamptic women and about 20% of preeclamptic women with neurological symptoms (Liman et al., 2012).

Regarding the long-term complications of preeclampsia, some authors have agreed that the white matter injuries observed on magnetic resonance imaging (MRI) several years after delivery, could be related to a higher risk of dementia or stroke (Aukes et al., 2012; Wiegman et al., 2014; Siepmann et al., 2017).

## The Blood-Brain Barrier in Preeclampsia

The BBB is a neurovascular unit that separates the brain tissue from systemic circulation. The brain capillary endothelial cells are the main element that forms the basic unit of BBB, along with the astrocytes, pericytes, and the adjacent neurons (Cantrill et al., 2012; Daneman and Prat, 2015). The endothelial cells join through strong tight junction proteins and substance transport is highly restricted due to the above feature and expression of membrane transporters belonging to the ATP-binding cassette and Solute Carrier families (Qosa et al., 2015; Morris et al., 2017).

Authors have proposed that the increased BBB permeability in preeclamptic pregnancies may be due to: (i) elevated microvascular pressure leading to vasogenic edema formation, (ii) alterations in expression/function of tight junction proteins, and (iii) circulating factors that increase the BBB permeability (i.e., altering transcellular transport) without modifying the mechanical barrier properties (Cipolla and Kraig, 2011).

To characterize the mechanisms involved in the cerebrovascular complications of preeclampsia, animal models have been vastly employed (Li et al., 2012; Warrington et al., 2014, 2015; Zhang and Warrington, 2016). However, in human, most of the research aiming to study the changes in the BBB functionality of preeclamptic women is carried out through MRI studies (Schwartz et al., 2000) and measurement of circulating levels of biomarkers of brain injury and endothelial damage (Bergman and Akerud, 2016; Bergman et al., 2016, 2018). Findings from these studies generally agree that preeclamptic women may suffer some degree of cerebrovascular damage. Recent studies published by our group showed that exposure of hCMEC/d3 cell monolayers (a human brain endothelial cell line) to plasma from preeclamptic pregnancies increased permeability to FITC-dextran 70 kDa and reduced their transendothelial electrical resistance (Bergman et al., 2021; Leon et al., 2021), with no changes in tight junction proteins mRNA expression (Bergman et al., 2021).

## Overview of VEGF Signaling and Its Role on the Cerebrovascular Complications of Preeclampsia

The VEGFs family is comprised of homodimeric proteins present in five different isoforms: VEGF-A, VEGF-B, VEGF-C, VEGF-D, and placental growth factor (PlGF) (Simons et al., 2016). These proteins, also known as angiogenic factors, are involved in vasculogenesis, vascular permeability (including brain circulation), nitric oxide synthesis stimulation, and endothelial cell survival. PlGF regulates the endothelial cell viability and the angiogenesis through its signaling pathways or amplifying VEGF-mediated actions (Schreurs et al., 2012).

The human VEGF receptors (VEGFRs) are tyrosine-kinase transmembrane receptors. Members of this family include VEGFR1 or Flt-1, VEGFR2 or KDR, and VEGFR3 or Flt-3. VEGFR2 have a higher tyrosine-kinase effect than Flt-1 and is the most relevant receptor regarding permeability and growth effects. VEGFR3 seems to participate in vascular development, but its primary function resides in the lymphatic vessels (Simons et al., 2016). VEGF presents an affinity for both, Flt-1, and VEGFR2 receptors, while PlGF binds only to Flt-1 (Schreurs et al., 2012). The soluble fraction of Flt-1 that contains the extracellular-ligand binding sites but lacks the intracellular and transmembrane sites, works as a decoy receptor that sequestrates VEGF and forms a heterodimer with VEGFR2, blocking the activation of the latter (Palmer et al., 2017).

Weeks before the onset of preeclampsia, the placenta secretes sFlt-1 (Levine et al., 2004; Chaiworapongsa et al., 2005). This outcome is thought to explain the elevated levels of this anti-angiogenic factor in the blood of preeclamptic women. sFlt-antagonize both VEGF and PlGF effects by reducing the circulating levels of the active forms, contributing to the clinical manifestations in preeclampsia (Maynard et al., 2003). In pregnant normotensive women there is a physiological increase in sFlt-1circulating levels and a reduction of PlGF between 33 and 36 weeks (Taylor et al., 2003), but in preeclampsia there is a loss of the balance between pro-angiogenic and anti-angiogenic factors that lead to the release of mediators from the placenta to the maternal circulation. This form of communication between the placenta and the maternal circulatory system is proposed to be responsible for the disruption of BBB, and the available evidence implies that not only sFlt-1 but VEGF, PlGF, pro-inflammatory cytokines and extracellular vesicles are also involved (Schreurs et al., 2012; Warrington et al., 2015; Clayton et al., 2018; Leon et al., 2021).

In preeclampsia, the imbalance between angiogenic and anti-angiogenic factors is proposed to play a key role in the increased cerebrovascular permeability (Bean et al., 2018). However, the exact mechanism by which the preeclampsia-mediated alterations in VEGF signaling modify the integrity of the BBB remain poorly known. Physiologically, it is well-documented that VEGFs cause transient opening of endothelial cell-cell junctions and VEGFR2 seems to be the main regulator of cell permeability. Some of the elucidated pathways for the increased permeability involve VEGFR2 signaling toward the Rous sarcoma homology 2-domain (Src) mediated by the T cell-specific adaptor (TSAd) (Sun et al., 2012), eNOS/NO mediated permeability (Fukumura et al., 2001), and caveolin-1 regulation of VEGF-signaling (Lin et al., 2007).

In the context of preeclampsia, a study demonstrated that plasma from preeclamptic pregnancies increased the permeability of cerebral rat veins (Amburgey et al., 2010), being this effect counteracted by co-treatment with a VEGFR2 inhibitor. The above outcome is confirmed in human hCMEC/d3 cells, as plasma from preeclamptic pregnancies, apart from increasing the permeability, up-regulated the mRNA expression of VEGFR2 and phosphorylation at the tyrosine residue Y951 (pY951), along with a decreased phosphorylation at the Y1175 (pY1175) residue (Bergman et al., 2021). The above findings agree with those that relate the pY951 with increased endothelial permeability (Matsumoto et al., 2005).

## Is the Endothelial Dysfunction at the BBB in Preeclampsia a Result of High sFlt-1 Levels?

It is well-known that preeclamptic women exhibit higher sFlt-1 levels, and this outcome is correlated to endothelial dysfunction (Maynard et al., 2003). The seminal work of Maynard et al. (2003), in which pregnant and non-pregnant mice injected with an adenovirus expressing sFlt-1, demonstrated that high levels of this protein are sufficient to elicit hypertension and glomerular endotheliosis. Further studies proposed a vicious cycle between sFlt-1, hypoxia and oxidative stress in the context of preeclampsia (Karumanchi and Bdolah, 2004). For example, mice exposed to exogenous sFlt-1 showed hypoxia and oxidative stress in the trophoblast, which caused more secretion of sFlt-1 (Jiang et al., 2015). A recent report shown that HUVECs treated with sFlt-1 exerted a pro-apoptotic effect by triggering the mitochondrial apoptosis pathway (Zhai et al., 2020). This hypothesis of the pathophysiology of preeclampsia has led to the development of strategies to restore the angiogenic balance. The removal of sFlt-1 from the circulation by apheresis has shown promising results as a potential treatment of preeclampsia, since increases the levels of free VEGF and PlGF in serum samples of preeclamptic patients (Matin et al., 2020).

However, it must be stressed that sFlt-1 is not the sole responsible of the anti-angiogenic imbalance in preeclampsia. Soluble endoglin (sEng), an anti-angiogenic protein that acts inhibiting the activity of transforming growth factor β1 (TGFβ1) (Levine et al., 2006), is also a contributing mediator. Studies have proposed that high levels are related to the severity of this syndrome as observed in women developing hemolysis elevated liver enzymes low platelets (HELLP) syndrome (Venkatesha et al., 2006), late-onset preeclampsia (Cim et al., 2017), and animal models of HELLP (Bean et al., 2018). TGFβ1 appears to modulate VEGFR2 signaling in endothelial cells, which results on the loss of both tip cell and stalk cell phenotypes (Jarad et al., 2017). However, its role on permeability needs to be further investigated.

**FIGURE 1:**
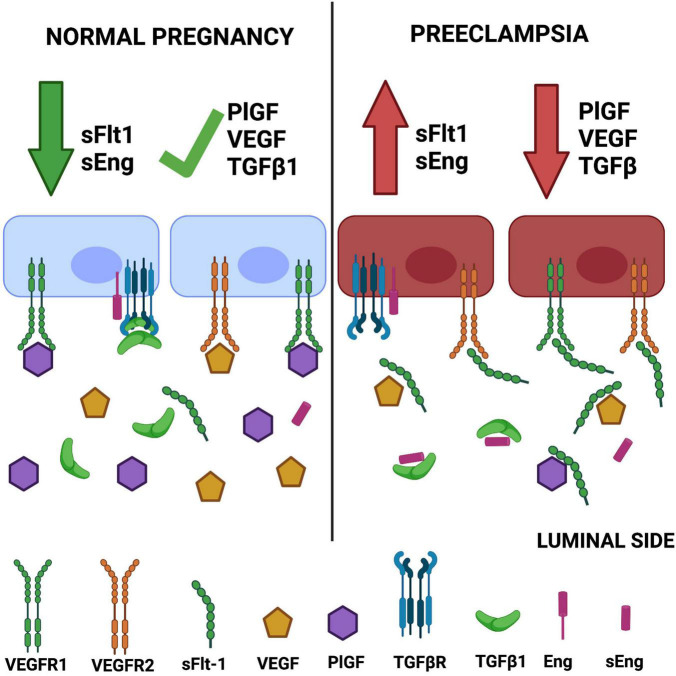
Angiogenic imbalance in preeclampsia. The classic hypothesis of angiogenic imbalance proposes that circulating levels of soluble tyrosine kinase-1 similar to fms (sFlt-1) and soluble endoglin (sEng) are increased in preeclampsia, reducing the concentrations of free vascular endothelial growth factor (VEGF)/placental growth factor (PlGF) and TGFβ, respectively. This outcome leads to endothelial dysfunction. Created with BioRender.com.

The above findings are consistent with reports demonstrating that treatment of HUVECs with plasma from preeclamptic women causes morphological changes, reduces cell proliferation, elicits mitochondrial damage, increases the permeability, and promotes apoptosis (Wu et al., 2012; Gao et al., 2016). A graphic description of the angiogenic imbalance in preeclampsia is presented in Figure 1.

According to the presented evidence, it appears that sFlt-1 can exert endothelial dysfunction by itself or in combination with sEng. However, as preeclampsia can lead to cerebrovascular complications without proper management, a group of studies conducted in rodent models attempted to characterize the effects of sFlt-1 in the brain vasculature. Surprisingly, a report demonstrated that overexpression of sFlt-1 in adult mice does not elicit significant changes in BBB permeability (Maharaj et al., 2008). However, mice overexpressing sFlt-1 and sEng exhibited increased BBB permeability (Maharaj et al., 2008). In a model of HELLP syndrome, induced by chronic administration of sFlt-1 and sEng to pregnant mice, a regional increase in BBB permeability at the posterior cortex was observed (Bean et al., 2016, 2018). Since the administration of exogenous sFlt-1/sEng leads to the development of a HELLP-like syndrome, other models such as the reduced utero-placental perfusion (RUPP) (Li et al., 2012) could be more suitable to study the cerebrovascular complications of preeclampsia. The RUPP model increases sFlt-1/sEng levels (Gilbert et al., 2007, 2009; Sun et al., 2020; Saif et al., 2021) and the permeability of the BBB (Warrington et al., 2014), but none of the presented evidence has proposed a mechanism by which these anti-angiogenic factors could be involved in the cerebrovascular complications elicited by this condition.

The above findings reinforce the hypothesis that the anti-angiogenic imbalance in preeclampsia has a peripheral source and requires a combination of factors to induce endothelial dysfunction at the BBB. However, in other pathological conditions involving the participation of VEGF signaling, the outcomes are contrasting. For example, in stroke (also a cerebrovascular complication of preeclampsia) the acute release of VEGF is known to increase BBB permeability (Geiseler and Morland, 2018). A study conducted in patients with subarachnoid hemorrhage demonstrated that under delayed tissue ischemia, the levels of VEGF and sFlt-1 increase in plasma/serum and cerebrospinal fluid (CSF) (Scheufler et al., 2003). The authors suggested that the source of higher VEGF and sFlt-1 levels in *cis*ternal CSF and systemic circulation comes from the local release from endothelial cells and glioneuronal secretion, and thrombocyte aggregations in ischemic brain regions, respectively. A recent study from our group demonstrated that patients with a first-ever ischemic stroke exhibited high circulating levels of VEGF, shortly after the ischemic event. This outcome is associated with a poor prognosis at the sixth month (Escudero et al., 2021).

In rodent models of acute brain ischemia/reperfusion, the gene transfer of sFlt-1 into the lateral ventricle reduced infarction, edema and BBB permeability (Kumai et al., 2007). The authors suggested that the acute effects of VEGF in BBB permeability were attenuated through reduction in the phosphorylation of focal adhesion kinase but did not provided findings to support this hypothesis. This protective effect of sFlt-1 on VEGF-induced BBB permeability has also been demonstrated in cerebral rat veins co-treated with exogenous sFlt-1 and plasma from late-pregnant rats, which exhibit higher levels of sFlt-1 (Schreurs et al., 2012).

According to the presented evidence, sFlt-1 exerts deleterious or protective effects on the brain vasculature depending on the type of insult applied. In preeclampsia, the hypothesis of endothelial dysfunction mediated by an imbalance between pro- and anti-angiogenic factors is often discussed assuming that in pregnancy, BBB endothelial cells can adapt to changes in blood VEGF levels through an increase in sFlt-1 as compensatory mechanism. However, this assumption can be misleading as in murine brain endothelial cells, the expression of VEGFR2 is polarized with higher expression in the basolateral side, while VEGFR1 is more expressed in the luminal side, contrary to the observed in lung endothelial cells (Hudson et al., 2014). Furthermore, this expression pattern is reflected by the formation of homodimers of each receptor, and presence of VEGFR1/2 heterodimers could be a result of the expression of residual basal VEGFR1 and luminal VEGFR2 (Hudson et al., 2014).

This compartmentalization of VEGF signaling BBB endothelial cells is important to understand their reactivity as they are exposed to autocrine and paracrine secretion of VEGF from neurons and astrocytes (Ogunshola et al., 2002), especially under hypoxic and inflammatory conditions (Liu et al., 2020) as observed in pathological conditions such as stroke (Li et al., 2014) and multiple sclerosis (Argaw et al., 2012; Chapouly et al., 2015). For instance, under hypoxic conditions, the release of VEGF in brain cells is regulated by the hypoxia inducible factor-1α (HIF-1), since the *VEGF* gene is activated by this transcription factor (Hu et al., 2003). Furthermore, pro-inflammatory cytokines including interleukin-1β activate pathways involved in the release of VEGF (Nagineni et al., 2012). In this regard, as ischemic and haemorrhagic stroke are cerebrovascular complications of preeclampsia (McDermott et al., 2018; Too et al., 2018), the paracrine and autocrine activation of VEGFR2 could be responsible of the increased BBB permeability. Indeed, studies have demonstrated that the genomic silencing of VEGF expression in astrocytes abrogates the breakdown of the BBB (Argaw et al., 2012). Therefore, the effects of sFlt-1 on VEGFR2 activity would be in theory protective and evident if the former crosses the BBB or if it is secreted in an autocrine manner (Ahmad et al., 2011). VEGFR1 is not directly involved in BBB permeability, but apparently plays a cytoprotective role in brain endothelial cells (Hudson et al., 2014).

**FIGURE 2:**
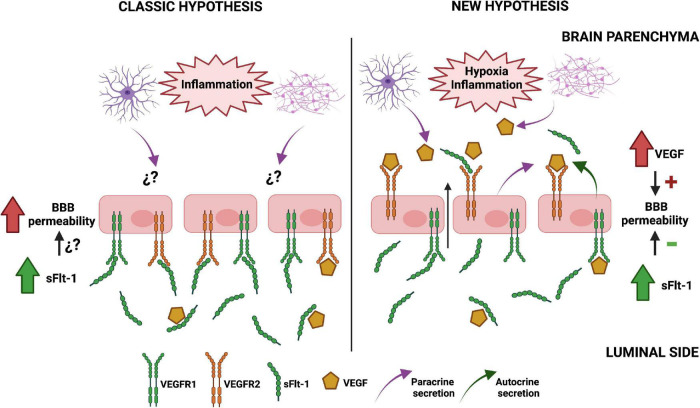
Proposed mechanism of soluble tyrosine kinase-1 similar to fms (sFlt-1) effects on blood-brain barrier (BBB) integrity in preeclampsia. The classic model of endothelial dysfunction in preeclampsia (left) assumes that the alterations on BBB functionality mainly depend on high circulating levels of anti-angiogenic factors such as sFlt-1, as the expression of VEGFRs is similar to the observed in peripheral endothelial cells. However, our hypothesis (right) proposes that the basolateral expression of VEGFR2 in brain endothelial cells makes them more reactive to the paracrine secretion of VEGF from astrocytes and neurons under pro-inflammatory conditions, increasing BBB permeability. Therefore, the high circulating levels of sFlt-1 in the blood of preeclamptic women and the autocrine secretion of this protein in brain endothelial cells might exert a protective effect. The figure omitted the inclusion of other relevant factors in order to better compare both hypotheses. Created with BioRender.com.

In summary, we propose a challenging hypothesis in which the compartmentalization of VEGF signaling in brain endothelial cells makes them more reactive to the paracrine secretion of this growth factor from brain cells in preeclampsia. As a result, the permeability of the BBB is increased and the high circulating levels of sFlt-1 observed in preeclamptic women may have a protective effect, which is not observed in peripheral endothelial cells such as HUVECs (Zhai et al., 2020). The Figure 2 presents a graphic description of this hypothesis.

This hypothesis, although plausible, is not without limitations. In our opinion, the lack of strong of evidence regarding the compartmentalization of VEGF signaling in peripheral endothelial cells would difficult the interpretation of findings. Further studies should focus their efforts in conducting comparative studies aiming to characterize the polarization of VEGFRs expression in human brain and peripheral endothelial cells. In addition, the multifactorial nature of preeclampsia suggests that the actions of sFlt-1 on the integrity of endothelial cell monolayers should be assessed under pro-inflammatory, hypoxic and anti-angiogenic environments.

Regarding the influence of inflammation on the pathophysiology of cerebrovascular complications of preeclampsia, neuroinflammation has gained relevance as an outcome that may help to explain certain long-term complications in women who had a preeclamptic pregnancy (Cipolla et al., 2012; Johnson et al., 2014; Clayton et al., 2018). The use of hypoxic conditions would prove useful to analyze the influence of HIF-1α on the reactivity of brain endothelial cells to the effects of sFlt-1. Lastly, the contribution of sEng and the TGF-β pathway to the anti-angiogenic component in preeclampsia’s pathophysiology is acknowledged, but despite this body of evidence, the effects at level of the brain vasculature require further characterization.

The above-described studies could be benefited using bi-cameral culturing systems such as Transwell© inserts, which allow co-culturing with other cell types including astrocytes and neurons. Furthermore, the use of human primary cultures or human-derived brain endothelial cell lines would provide more relevant findings or validate previous results collected from animal models. This knowledge will provide a deeper insight on the reactivity of the BBB to pro- and anti-angiogenic conditions. As most of the presented evidence does not always associate the effects of sFlt-1 on the dynamics of VEGFRs function and its downstream events, this gap in knowledge represents an opportunity to better characterize its actions on pathologies including preeclampsia.

## Concluding Remarks

In the last two decades, significant advances have been achieved on the study of the cerebrovascular complications of preeclampsia. There is a better understanding of its pathophysiology, and more recently, this knowledge is being applied to characterize the long-lasting effects of this syndrome on cognition. However, there are many questions to be answered regarding the communication between the placenta and the maternal brain, and the reactivity of brain endothelial cells to noxious circulating factors in preeclamptic women.

The influence of sFlt-1 on VEGF signaling and the development of vascular dysfunction in preeclampsia has not been fully explored at a molecular level, despite its use as a routinary diagnostic biomarker in many countries including the United Kingdom. There is a need for more studies attempting to understand how sFlt-1 alters the dynamics of VEGFRs activation and the crosstalk with other pathways involved in angiogenesis and inflammation. Since most of the current knowledge has been generated from the use of non-cerebral endothelial cell models, we encourage further research in the brain vasculature. If the increase on sFlt-1 levels may eventually be protective instead of harmful factor for the BBB, this knowledge will not only bring more understanding of the pathophysiology of brain complications in preeclampsia, but would help to discovery new therapeutic targets. In particular, the inhibition of VEGFR2 can revert the BBB disruption induced with plasma of women with preeclampsia, but potential therapeutic applicability of these findings has not been even proposed in the literature.

## Data Availability Statement

The original contributions presented in the study are included in the article/supplementary material, further inquiries can be directed to the corresponding authors.

## Author Contributions

PT-V conceived and wrote the manuscript. RR contributed to the writing of the manuscript. CE contributed to the writing of the manuscript and provided a critical revision of the contents. All authors contributed to the article and approved the submitted version.

## Conflict of Interest

The authors declare that the research was conducted in the absence of any commercial or financial relationships that could be construed as a potential conflict of interest.

## Publisher’s Note

All claims expressed in this article are solely those of the authors and do not necessarily represent those of their affiliated organizations, or those of the publisher, the editors and the reviewers. Any product that may be evaluated in this article, or claim that may be made by its manufacturer, is not guaranteed or endorsed by the publisher.
